# Supplementary material for: Content Validity of a Scale Designed to Measure the Access of Older Adults to Outpatient Health Services
Source: Int J Environ Res Public Health. 2022 Aug 16;19(16):10102. doi: 10.3390/ijerph191610102 (PMC9407808; doi:10.3390/ijerph191610102)
Supplement: Supplementary file 1 [file ijerph-19-10102-s001.zip › Table S1_12agos22.pdf]

Table S1. Distribution of the background characteristics of the base sample of participants-judges (older adults) according to their reading comprehension *(*n=46).

	**Limited reading comprehension 23 (50%)**	**Adequate reading comprehension ± 23 (50%)**	***P*-value**
Sex			1.000
Men	10 (43.48)	10 (43.48)	
Women	13 (56.52)	13 (56.52)	
Marital status			0.615
Single	3 (13.04)	6 (26.09)	
Married	15(65.22)	12 (52.17)	
Widowed	5 (21.74)	5 (21.74)	
Schooling			0.554
Incomplete elementary school	12 (52.17)	8 (34.78)	
Complete elementary school	7 (30.43)	10 (43.48)	
Middle school and beyond	4 (17.39)	5 (21.74)	
Occupation			0.670
Unpaid domestic work	8 (34.78)	10 (43.48)	
Agricultural activities	5 (21.74)	3 (13.04)	
Commerce	7 (30.43)	5 (21.74)	
Others	3 (13.04)	5 (21.74)	
Health needs			
Yes	15 (65.22)	7 (30.43)	0.018*
No	8 (34.78)	16 (69.57)	
Care seeking			
Yes	15 (65.22)	8 (34.78)	0.039*
No	8 (34.78)	15 (65.22)	
	*Median (IQR** 25-75)*	*Median (IQR** 25-75)*	0.808
Age (years)	65 (62-71)	67 (64-72)	

Note: *Association between categorical variables was tested using the Chi-square test or Fisher's exact test: *p* <0.05
**IQR: Interquartile range. The median with IQR range was compared using the Mann Whitney U test: *p* <0.05
± Effective population: Participants-judges included in the content validity analysis.
