# Supplementary material for: Content Validity of a Scale Designed to Measure the Access of Older Adults to Outpatient Health Services
Source: Int J Environ Res Public Health. 2022 Aug 16;19(16):10102. doi: 10.3390/ijerph191610102 (PMC9407808; doi:10.3390/ijerph191610102)
Supplement: Supplementary file 1 [file ijerph-19-10102-s001.zip › Table S2_12agos22.pdf]

Table S2

Escala de acceso de personas mayores a servicios de salud ambulatorios de primer nivel (*Access of Older adults to Outpatient Primary-Care Health Services Scale, AOAOPHSS*).

El acceso a la atención de salud es consecuencia de la integración que se da entre diversos determinantes relacionados con las habilidades de las personas (capacidad de percepción, búsqueda, pago, etc.) y las diferentes características de los servicios de atención (disponibilidad, asequibilidad, adecuación, etc.). La siguiente escala se diseñó para ayudar a comprender la naturaleza integral y dinámica del acceso de personas mayores a los servicios ambulatorios de primer nivel.

Después de leer cuidadosamente los siguientes enunciados, deberá colocar una cruz en el cuadro del número que refleje mejor el grado de acuerdo respecto a cada oración señalada, tomando en cuenta que el número 1 indica *en desacuerdo* y al avanzar la numeración aumenta el grado de acuerdo hasta llegar al máximo que es el número 3, *de acuerdo*.

En desacuerdo	**1**	**2**	**3**	*De acuerdo*

**I.-SUBESCALA DE ACCESIBILIDAD**				
**A) ABORDABILIDAD**				
**1**	Los servicios de salud informan la situación de salud en la que se encuentra mi comunidad	**1**	**2**	**3**
**2**	Los servicios de salud informan los resultados de las supervisiones que a ellos les hacen	**1**	**2**	**3**
**3**	Los servicios de salud informan sobre el compromiso que tienen con mi comunidad	**1**	**2**	**3**
**4**	Los servicios de salud se dan a conocer entre la gente de mi comunidad	**1**	**2**	**3**
**5**	Es fácil ubicar a los servicios de salud de mi comunidad	**1**	**2**	**3**
**6**	Los servicios de salud informan sobre los servicios de atención que ofrecen en mi comunidad	**1**	**2**	**3**
**7**	Los servicios de salud a menudo realizan campañas de salud en mi comunidad	**1**	**2**	**3**
**8**	Los servicios de salud reparten folletos y carteles para el cuidado de la salud	**1**	**2**	**3**
**B) ACEPTABILIDAD**				
**9**	Los servicios de salud tienen intérpretes para la atención de la gente que no habla español	**1**	**2**	**3**
**10**	Los servicios de salud dan la oportunidad de elegir si quiere que le atienda un hombre o una mujer	**1**	**2**	**3**
**11**	En los servicios de salud se respetan las costumbres de la gente	**1**	**2**	**3**
**12**	En los servicios de salud se respeta la religión de la gente	**1**	**2**	**3**
**C) DISPONIBILIDAD/ALOJAMIENTO**				
**13**	Existen servicios de salud en mi comunidad	**1**	**2**	**3**
**14**	Los servicios de salud están ubicados cerca de mi comunidad	**1**	**2**	**3**
**15**	La construcción de los servicios de salud está en buenas condiciones	**1**	**2**	**3**

**16**	El transporte público pasa cerca de los servicios de salud	**1**	**2**	**3**
**17**	Los espacios físicos de los servicios de salud son cómodos para las personas mayores	**1**	**2**	**3**
**18**	En los servicios de salud hay rampas para personas con dificultad para moverse	**1**	**2**	**3**
**19**	En los servicios de salud con más de un piso hay elevador para personas con dificultad para moverse	**1**	**2**	**3**
**20**	Los servicios de salud cuentan con suficiente personal para atender a las personas mayores	**1**	**2**	**3**
**21**	Los servicios de salud funcionan en las tardes	**1**	**2**	**3**
**22**	Los servicios de salud funcionan el fin de semana	**1**	**2**	**3**
**23**	Los servicios de salud programan la consulta mediante cita previa	**1**	**2**	**3**
**24**	La consulta en los servicios de salud se puede programar por teléfono	**1**	**2**	**3**
**25**	Se puede consultar al personal de los servicios de salud por teléfono	**1**	**2**	**3**
	**D) ASEQUIBILIDAD**			
**26**	La consulta en los servicios de salud es cara	**1**	**2**	**3**
**27**	Los medicamentos que recetan en los servicios de salud son caros	**1**	**2**	**3**
**28**	Los exámenes de laboratorio que piden en los servicios de salud son caros	**1**	**2**	**3**
**29**	Acudir a los servicios de salud implica gastar en alimentos	**1**	**2**	**3**
**30**	El traslado para ir a los servicios de salud es caro	**1**	**2**	**3**
**31**	Acudir a los servicios de salud implica perder ingresos	**1**	**2**	**3**
	**E) ADECUACIÓN**			
**32**	La atención que brinda el personal de los servicios de salud es buena	**1**	**2**	**3**
**33**	El personal de los servicios de salud aclara dudas sobre el tratamiento que receta	**1**	**2**	**3**
**34**	El personal de los servicios de salud dedica tiempo suficiente a la consulta	**1**	**2**	**3**
**35**	El personal de salud conoce el historial clínico de sus pacientes	**1**	**2**	**3**
**36**	El personal de salud da seguimiento al tratamiento de sus pacientes	**1**	**2**	**3**
**37**	El personal de los servicios de salud enseña a los pacientes a cuidar su salud	**1**	**2**	**3**
**38**	Los servicios de salud dan pases para ir con un especialista en caso de necesitarlo	**1**	**2**	**3**

**II.-SUBESCALA HABILIDADES PERSONALES**				
	**A) PERCEPCIÓN**			
**39**	Cuando tengo una necesidad de salud, prefiero ir con brujos o curanderos	**1**	**2**	**3**
**40**	Creo que los servicios de salud ayudan a mejorar la salud	**1**	**2**	**3**
**41**	Me da miedo acudir a los servicios de salud	**1**	**2**	**3**
**42**	Pienso que mis problemas de salud pueden mejorar por sí solos	**1**	**2**	**3**
**43**	Tengo muchas dudas sobre la atención que dan en los servicios de salud	**1**	**2**	**3**
	**B) BÚSQUEDA**			
**44**	Tengo derecho a que me atiendan en los servicios de salud	**1**	**2**	**3**
**45**	Me es fácil buscar información sobre los diferentes servicios de salud	**1**	**2**	**3**
**46**	Cuando necesito de un servicio de salud me es fácil decidir a dónde ir	**1**	**2**	**3**
	**C) ALCANCE**			
**47**	Tengo facilidad para ir a los servicios de salud cuando los necesito	**1**	**2**	**3**
**48**	Mi condición física me impide ir a los servicios de salud	**1**	**2**	**3**
**49**	Cuento con servicio de transporte para ir a los servicios de salud	**1**	**2**	**3**
**50**	El transporte para ir a los servicios de salud es adecuado para mí	**1**	**2**	**3**
	**D) PAGO**			
**51**	Cuento con un trabajo por el cual recibo un salario	**1**	**2**	**3**
**52**	Recibo dinero por la pensión de mi antiguo trabajo	**1**	**2**	**3**
**53**	Recibo dinero por parte del gobierno	**1**	**2**	**3**
**54**	Tengo cuentas de ahorro o inversiones a plazo fijo en algún banco	**1**	**2**	**3**
**55**	Tengo propiedades a mi nombre	**1**	**2**	**3**
	**E) COMPROMISO**			
**56**	Me comprometo con la atención de mi salud	**1**	**2**	**3**
**57**	Si en poco tiempo veo mejoría en mi salud dejo los tratamientos	**1**	**2**	**3**
**58**	Si tengo que seguir una dieta la respeto	**1**	**2**	**3**
**59**	Participo en la toma de decisiones sobre el tratamiento que me ofrecen los servicios de salud	**1**	**2**	**3**
**60**	Cuando estoy en los servicios de salud participo al explicar mis necesidades de salud	**1**	**2**	**3**

¡Gracias por sus respuestas!
